# Comparative Analysis of Flavor and Starch Physicochemical Properties in Different Varieties of Baked Sweet Potatoes

**DOI:** 10.3390/foods15050802

**Published:** 2026-02-24

**Authors:** Wen Li, Chunjie Zhang, Huijun Cui, Siguo Xiong, Hui Xie, Chenghui Liu, Chen Chen, Aili Jiang

**Affiliations:** 1 College of Life Sciences, Dalian Minzu University, Dalian 116600, China; lwen_2026@163.com (W.L.); m18941355962@163.com (C.Z.); chj0423@163.com (H.C.);; 2 Key Laboratory of Biotechnology and Bioresources Utilization, Ministry of Education, Dalian 116600, China; 3 Dalian Special Cereals Research Institute, Dalian 116200, China

**Keywords:** sensory evaluation, volatile organic compounds, sugar content, starch physicochemical properties

## Abstract

This study aimed to investigate the flavor quality and starch physicochemical properties of three orange-fleshed sweet potato varieties commonly cultivated in northeastern China. Fresh and baked samples were evaluated using sensory analysis, electronic nose and tongue, gas chromatography-mass spectrometry for volatile compound profiling, and chemical methods for starch characterization. Liankaoshu 1 exhibited the highest sensory score (88.6), reflecting superior taste and aroma. A total of 70 volatile organic compounds were identified, including β-damascenone, maltol, and β-ionone, as key contributors to baked flavor. Significant varietal differences were found in starch content, particle size, and crystalline structures, with Pushu 32 showing CA-type crystals, Yanshu 25 A-type, and Liankaoshu 1 B-type. Baking increased maltose and soluble sugar levels, which were strongly correlated with sensory attributes. Spearman correlation analysis revealed that sweetness and overall sensory scores were significantly and positively correlated with maltose, soluble sugar, and reducing sugar contents, as well as starch particle size parameters (*p* ≤ 0.05). These results indicate that starch structural characteristics and saccharification efficiency play critical roles in regulating flavor formation during baking, providing a theoretical basis for sweet potato breeding and processing optimization.

## 1. Introduction

Sweet potato (*Ipomoea batatas* (L.) Lam.) is a globally significant crop known for its versatility, yet its full potential remains underutilized [1]. In 2023, the global output of sweet potatoes was around 93.52 million tons, with China accounting for 55% of the total [2]. Sweet potato is not only a traditional staple crop but also regarded as an important source of carbohydrates (20.12 g·100 g^−1^), protein (1.57 g·100 g^−1^), and minerals (0.99 g·100 g^−1^) [3]. In addition, it offers various health benefits, including antioxidant activity [4], immune system enhancement [5], and assistance in preventing and managing cardiovascular diseases [6], thus contributing significantly to public health improvement [7]. The increasing awareness of these benefits has led to a rise in sweet potato consumption worldwide [8]. Sweet potatoes are typically consumed after cooking, with common methods including boiling, steaming, baking, and frying. Among these, baked sweet potatoes are particularly popular in northeastern China due to their unique flavor. Compared with other cooking methods, baking triggers Maillard and caramelization reactions, producing a variety of volatile organic compounds (VOCs) and soluble sugars, resulting in an appealing color and rich flavor [5].

Key quality parameters for evaluating baked sweet potatoes include appearance, texture, aroma, and taste. Research has demonstrated significant changes in the color, flavor compounds, and sugar composition of sweet potatoes after baking, which directly influence their overall acceptability [9]. The taste profile of baked sweet potatoes includes sourness, sweetness, bitterness, umami, and astringency [10], among which sweetness is the most important characteristic and plays a decisive role in consumer purchasing decisions [11]. The sweetness of baked sweet potatoes primarily results from the saccharification process at high temperatures, where varieties with high saccharification efficiency exhibit enhanced sweetness and superior sensory scores after baking [12]. During baking, starch degrades into soluble sugars, leading to a softer texture and intensified sweetness [13]. Studies have shown that raw sweet potatoes contain negligible maltose, but its content increases significantly after cooking [12,14,15]. For example, Jiang et al. reported that steaming increased the maltose content of purple-fleshed sweet potatoes from 0.59 μg g^−1^ to 123.65 μg g^−1^ [7]. Given the strong positive correlation between maltose content and sweetness, maltose is considered the primary contributor to the sweetness of sweet potatoes [8,14]. However, maltose synthesis is influenced by complex physiological and biochemical processes, including the activity of starch-degrading enzymes and starch structural properties [14,15]. Additionally, saccharification efficiency is affected by genotype, growth environment, and storage conditions [12,16,17,18].

Historically, sweet potato varieties cultivated in China were categorized as “moderately sweet,” significantly less sweet than the “extremely sweet” varieties common in the United States [19]. At that time, most Chinese sweet potato resources were primarily used for starch processing, and high-saccharification-efficiency varieties suitable for baking were scarce. In recent years, however, rising living standards and advances in breeding technology have led to the development of numerous high-quality sweet potato varieties in China, meeting consumer demands for improved flavor and nutritional benefits [20]. Sweet potatoes are typically classified by flesh color into white, yellow, orange, and purple varieties, each suitable for specific cooking methods [21]. Among these, orange-fleshed varieties are particularly favored for baking due to their superior flavor and sweetness [13]. However, substantial differences in flavor may still exist within the same flesh color group due to variations in starch granule size, starch composition, and molecular and crystalline structures among different genotypes [22]. Understanding these varietal differences is essential for satisfying diverse consumer preferences and expanding market opportunities.

While prior research has investigated the flavor and chemical profiles of sweet potatoes with various flesh colors, in-depth comparative analyses specifically targeting high-quality orange-fleshed varieties are still scarce. This study hypothesizes that the physicochemical properties of starch play a pivotal role in regulating flavor and texture, contributing to significant sensory quality differences among high-quality orange-fleshed sweet potato varieties. This study aims to identify key physicochemical indicators that influence the sensory quality of baked sweet potatoes, offering valuable insights for the development of sweet potato germplasm resources and the optimization of baking processes.

## 2. Materials and Methods

### 2.1. Raw Materials

P32 is a high-quality sweet potato variety developed by the Puning Agricultural Science Research Institute, known for its high yield, stable productivity, and excellent output potential. Y25, bred by the Sweet Potato Research Institute of the Yantai Academy of Agricultural Sciences, is characterized by its high yield and strong disease resistance, making it one of the most popular baked sweet potato varieties in the Chinese market in recent years. LK1 is a newly developed baked sweet potato variety from the Dalian Special Grain Research Institute, distinguished by its strong stress resistance, fine texture, high sweetness, and low fiber content. The P32 and Y25 samples were purchased as freshly harvested products from a local produce supermarket in Jinzhou District, Dalian. LK1 samples were provided by the Agricultural Ecological Base of the Dalian Special Cereals Research Institute. All samples were from the 2024 harvest season and were stored at 23 ± 2 °C and 85% relative humidity prior to analysis. Due to the different sources, exact cultivation duration and harvest time could not be fully unified. Therefore, this study focused on exploring the relative differences in starch degradation and physicochemical properties among varieties under real-world conditions rather than strictly standardized agronomic comparisons.

### 2.2. Cooking Procedure and Sample Preparation

Uniform, spindle-shaped sweet potatoes weighing 150–200 g, and free from disease and mechanical damage, were chosen. The tubers were thoroughly rinsed with tap water, air-dried, and then randomly assigned to two groups. One group was retained as raw samples, while the other group was placed on the middle stainless steel rack of a preheated oven at 200 °C (PT25 × 1, Midea, Beijing, China), with approximately 5 cm spacing between samples to ensure even heating. The sweet potatoes were baked for 90 min under these conditions, following the procedure reported by Zhang et al. [8].

Following baking, the samples were promptly taken out and cooled to room temperature. Both raw and baked sweet potatoes were peeled, and the central portion was collected for further analysis. A portion of the sample was used fresh for electronic nose, electronic tongue analysis, and sensory evaluation. The remaining samples were cut into small pieces and ground into powder under liquid nitrogen. The resulting powder was split into two portions: one was stored at −80 °C, and the other was freeze-dried for 48 h, passed through a 200-mesh sieve, and kept in sealed bags at −20 °C for subsequent analysis.

### 2.3. Determination of Moisture and Dry Matter Content

Moisture and dry matter content were quantified by the freeze-drying method following Pinhero et al. [23]. The calculations were performed using the following formulas.

(1) $$Moisture\;content\;\left(\%\right)=\frac{W_{1}-W_{2}}{W_{1}}\times 100$$

(2) $$Dry\;matter\;content\;\left(\%\right)=\frac{W_{1}}{\;W_{2}}\times 100$$

where *W*_1_ represents the weight of the frozen fresh powder, and *W*_2_ represents the weight of the freeze-dried powder.

### 2.4. Measurement of Odor and Taste Using Electronic Nose and Tongue

Sweet potato odor characteristics pre- and post-baking were evaluated with a PEN3 portable electronic nose (INSENT Company, Atsugi, Japan) following Xu et al. [24]. Fresh samples (5 g) were loaded into 20 mL headspace vials, sealed, and allowed to equilibrate at room temperature for 30 min. Volatile compounds were collected using a headspace sampling method. The parameters were set as follows: air purging time of 60 s, sample testing time of 60 s, and airflow rate of 400 mL min^−1^. The odor response values at 56–58 s were used for data analysis. All samples were measured under identical experimental conditions using the electronic nose, with each sample subjected to three replicate measurements (*n* = 3) to confirm the repeatability and stability of the detection. The characteristics of the electronic nose sensors are detailed in Supplementary Table S1.

The taste properties, including sweetness, sourness, bitterness, umami, saltiness, and astringency, were assessed using the SA402B electronic tongue (INSENT Company, Atsugi, Japan) as described by Xiong et al. [25]. In brief, 20 g of fresh sample was homogenized with 100 mL of deionized water, followed by centrifugation at 8000× *g* for 15 min at 25 °C. The supernatant was transferred to a sample cup for measurement. During the detection process, the sweetness sensor was set to five cycles, while all other sensors were set to four cycles.

### 2.5. Sensory Evaluation

The sensory evaluation method was modified based on the work of Bao et al. [26] and Yao et al. [27]. The evaluation panel consisted of 16 trained panelists (8 males and 8 females, aged 20–45 years). All members received at least 90 h of professional training to ensure their ability to match, evaluate flavors, and accurately describe the sensory characteristics of roasted sweet potatoes. The evaluation focused on six key attributes: firmness, starchiness, viscosity, fibrous texture, aroma, and sweetness. The evaluation criteria (Supplementary Table S2) were developed and agreed upon by the panelists during the training period.

Baked sweet potato samples were cut into 2 cm cubes at room temperature for evaluation. During the assessment, panelists were instructed not to communicate or swallow the samples. After each evaluation, panelists rinsed their mouths with purified water to remove any residual taste and restore sensory neutrality. The scoring system assigned a maximum of 20 points each for aroma and sweetness, while the other attributes were scored on a 15-point scale. The total sensory score was calculated as the sum of all attribute scores, with a maximum score of 100.

The roasted sweet potato samples were prepared using simple cooking methods and pose no adverse health effects. All participants provided informed consent and retained the right to unconditionally withdraw from the study at any time. This research is non-commercial in nature and does not collect sensitive personal data. All collected data have been anonymized. Participants were all adults and were fully informed of the study content and their rights. In accordance with Article 32 of the “Ethical Review Measures for Life Sciences and Medical Research Involving Humans” (Document No. 4) issued by the National Health Commission of China in 2023, this study meets the criteria for exemption from ethical review.

### 2.6. Determination of VOCs

The method described by Xu et al. [24] was modified to analyze the aroma components and their relative contents in different sweet potato varieties before and after baking using headspace solid-phase microextraction (HS-SPME) coupled with GC-MS. A precise 2 g of frozen powder was homogenized with 4 mL saturated sodium chloride solution in a headspace vial and sealed. Raw and baked sample mixtures were incubated at 40 °C and 70 °C for 30 min, respectively. Volatile compounds were subsequently extracted using an SPME fiber (Supelco, Inc., Bellefonte, PA, USA) for 30 min at the same temperatures.

The captured VOCs were desorbed at 250 °C for 3 min and separated using an Rxi-5Sil MS column (30 m × 0.25 mm × 0.25 μm, Agilent Technologies, Santa Clara, CA, USA). The initial column temperature was set at 35 °C and held for 2 min, followed by an increase at 6 °C min^−1^ to 160 °C, and then ramped at 10 °C min^−1^ to 250 °C. Helium was used as the carrier gas at a constant flow rate of 1 mL min^−1^. The system operated in electron impact mode at 70 eV, with interface and ion source temperatures of 230 °C and 200 °C, respectively. Mass spectra were acquired in full scan mode over a range of *m*/*z* 50–500. Volatile compounds were identified by comparing their mass spectra with the NIST 17 mass spectral library, combined with comparison of their calculated retention indices (RI) with published data. The RI values were calculated using a C7–C30 n-alkane mixture analyzed under the same GC–MS conditions as the samples. The relative content of each volatile compound was determined by area normalization. Only compounds with a mass spectral similarity index higher than 80 and reasonable RI agreement were considered to be positively identified.

### 2.7. Determination of Soluble Sugar, Reducing Sugar, Sucrose, Maltose, Fructose, Glucose Contents, and Sweetness Value

The soluble sugar content was determined using the phenol-sulfuric acid method. Briefly, 1 g of frozen fresh powder was homogenized in distilled water and extracted in a boiling water bath for 20 min. The filtrate was collected (extraction repeated twice). Then, 0.5 mL of the crude extract was mixed with 1.5 mL of distilled water, followed by the sequential addition of 1.0 mL phenol solution (0.09 g mL^−1^) and 0.5 mL concentrated sulfuric acid (specific gravity 1.84). Following thorough mixing, the reaction was carried out at room temperature for 30 min. Absorbance was measured at 485 nm, and a standard curve was prepared using sucrose.

The 3,5-dinitrosalicylic acid (DNS) method was used to determine reducing sugar content. Absorbance was measured at 540 nm, and glucose was used to establish the standard curve [28].

The contents of sucrose, fructose, glucose and maltose, were quantified using commercial assay kits (Comin Biotechnology Co., Ltd., Suzhou, China). The absorbance values at 480 nm (sucrose, fructose, glucose) and 505 nm (maltose) were determined respectively. All sugar contents were expressed on a fresh weight (FW) basis as g kg^−1^.

The sweetness value was calculated based on the sweetness Indices proposed by Mao et al. [29]. The sweetness indices for sucrose, maltose, fructose, and glucose are 1.00, 1.25, 0.94, and 1.12, respectively. The formula for calculating sweetness value is as follows:

Sweetness value = 1.00 × sucrose content + 1.25 × maltose content + 0.94 × fructose content + 1.12 × glucose content

### 2.8. Determination of Starch, Amylose, Rapidly Digestible Starch (RDS), Slowly Digestible Starch (SDS), and Resistant Starch (RS)

The starch content was determined using the acid hydrolysis method described by Cao et al. [30]. The glucose content in the extract was measured using the DNS method, and the starch content was calculated and expressed as g kg^−1^ FW.

Amylose content was determined using its ability to form a blue complex with iodine. A commercial assay kit (Comin Biotechnology Co., Ltd., Suzhou, China) was used to measure amylose in freeze-dried powder, and the results were expressed as g kg^−1^ dry weight (DW).

RDS, SDS, and RS were evaluated using a modified method described by Sun et al. [31]. Freeze-dried powder (0.2 g) was homogenized in 15 mL of 0.1 mol L^−1^ sodium acetate buffer (pH 5.2) and incubated at 90 °C for 10 min to achieve gelatinization. After cooling to 37 °C, 10 mL of an enzyme mixture (containing 700 mg porcine pancreatic α-amylase (60 U mg^−1^) and 15 U mg^−1^ glucoamylase) was added. The mixture was shaken at 50 rpm/min in a water bath at a constant temperature for 2 h to simulate in vitro digestion. Aliquots (1 mL) were taken at 0, 20, and 120 min, mixed with 8 mL of ethanol to terminate the reaction, and centrifuged. The digestion rate was determined using the DNS method and calculated as follows:

(3) $$RDS\;(\%)\;=\;\frac{\left(G_{20}-FG\right)\;\times \;0.9}{TS}\;\times \;100$$

(4) $$SDS\;(\%)=\frac{\left(G_{120}-G_{20}\right)\times 0.9}{TS}\times 100$$

(5) RS (%)=100%−RDS−SDS

where *G*_20_ and *G*_120_ represent the glucose content at 20 and 120 min of starch hydrolysis, respectively; *FG* is the free glucose content, and *TS* is the total starch content.

### 2.9. Extraction of Starch Granules

Starch granules were extracted using the water extraction method with slight modifications [32]. Specifically, the sweet potato storage roots were washed, peeled, and cut into small pieces (2–3 cm). The pieces were blended with water at a ratio of 1:4 (*w*/*v*) using a blender (JYL-C23, JOyoung, Hangzhou, China) to fully release the starch granules. The resulting sweet potato slurry was filtered through a 100-mesh sieve. The filtrate was allowed to settle for 6 h, after which the supernatant was discarded, and the precipitate was retained. The precipitate was washed repeatedly with water until the supernatant became clear (approximately five washes). The purified starch was dried at 40 °C for 2 d, ground into powder, and passed through a 200-mesh sieve.

### 2.10. Morphological Observation of Starch

Starch microstructure was analyzed via scanning electron microscope (SEM) (S-4800; Hitachi, Tokyo, Japan) following Li et al. [33]. The samples were dispersed onto conductive adhesive and fixed onto a specimen holder. Gold coating was applied under vacuum conditions for 30 s. Imaging was performed at an acceleration voltage of 5 kV with magnifications of 600× and 1000×.

### 2.11. Determination of Starch Particle Size Distribution

The starch particle size distribution was determined following the method described by Li et al. [33]. A laser diffraction particle size analyzer (Bettersize2600, Bettersize Instruments Ltd., Dandong, China) was used to measure particle size parameters, including the volume-based diameter (*D*_(4,3)_), surface-based diameter (*D*_(3,2)_), specific surface area (S.S.A.), and dispersion (calculated using the formula below):

(6) $$Dispersion\;=\;(D_{90}-D_{10})/D_{50}$$

Here, *D*_10_, *D*_50_, and *D*_90_ represent the particle diameters below which 10%, 50%, and 90% of the starch particles fall, respectively.

### 2.12. X-Ray Diffraction (XRD) Analysis of Starch Crystalline Structure

The crystalline structure of starch was analyzed using an XRD (XRD-6000, Shimadzu, Kyoto, Japan) following the method described by Sun et al. [31]. Briefly, starch samples were evenly distributed in the sample holder and analyzed under an accelerating voltage of 40 kV and a current of 40 mA. XRD patterns were recorded in the scanning range of 4° to 40° with a scan rate of 2° min^−1^ and a step size of 0.02°.

### 2.13. Fourier Transform Infrared Spectroscopy (FT-IR) Analysis of Starch Structure

The starch structure was analyzed using FT-IR (IRAffinity-1, Shimadzu, Kyoto, Japan) following the method described by Gong et al. [34]. Briefly, 1 mg of starch sample was mixed with 150 mg of potassium bromide (KBr), finely ground, and compressed into a pellet. The FT-IR spectra were recorded using KBr as the background, with a scanning range of 400 to 4000 cm^−1^, a resolution of 4 cm^−1^, and 64 scans to ensure high-quality spectral data.

### 2.14. Statistical Analysis

All experimental measurements were conducted with three or more biological replicates, and data were presented as mean ± standard deviation. Statistical analyses were performed using SPSS 24.0 software. Differences between raw and baked samples were assessed using an independent sample *t*-test. One-way ANOVA was used to analyze differences among different sweet potato varieties. Significant differences between treatment groups were then assessed using the Duncan Mean Separation Test and the LSD Test (*p* < 0.05). Correlations between variables were analyzed using Spearman’s correlation, and clustering heatmaps were generated with TBtools software Version 2.1. Other visualizations were created using Origin 2021 software.

## 3. Results and Discussion

### 3.1. Quality Characteristics Analysis

**Figure 1:**
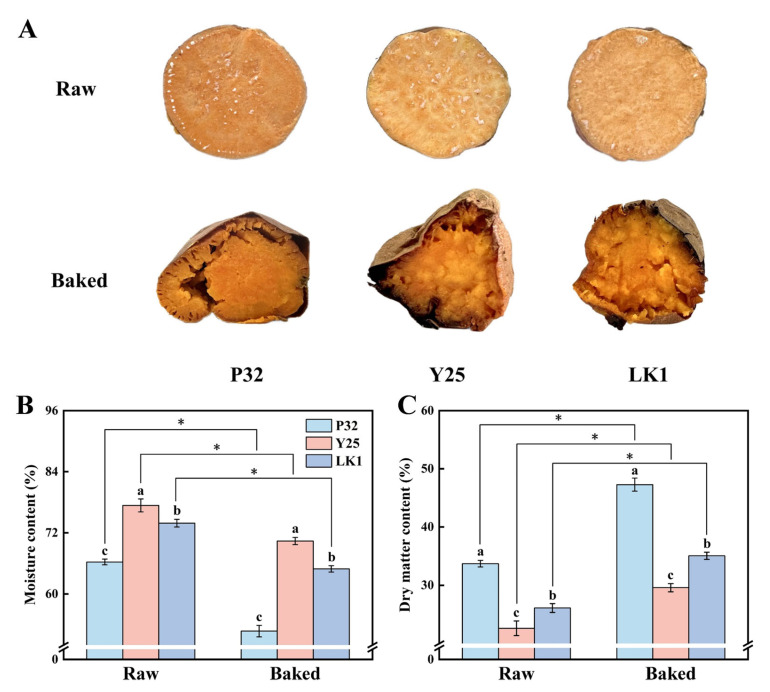
Quality characteristics of different varieties of sweet potatoes before and after baking. (**A**) Phenotypic changes; (**B**) Moisture content; (**C**) Dry matter content. P32: Pushu 32; Y25: Yanshu 25; LK1: Liankaoshu 1. Data were presented as mean ± standard deviation, the varieties were analysed by ANOVA and different lower case letters indicate significant differences between varieties (*p* < 0.05); differences between raw and baked samples were assessed using an independent sample *t*-test, * indicates that there was a significant difference at *p* < 0.05.

Figure 1A highlights the phenotypic changes in sweet potatoes after baking, characterized by a deepened color that significantly enhances their visual appeal. This transformation is likely driven by sucrose dehydration, thermal degradation, and the Maillard reaction, which collectively produce caramelized compounds and dark-colored products [13]. Baking led to notable weight loss in sweet potatoes, primarily due to moisture evaporation at high temperatures (Figure 1B,C). Among the varieties, baked P32 exhibited the lowest moisture content (52.73%), followed by LK1 (64.94%), whereas the dry matter content showed an opposite trend (P32 > LK1 > Y25). The decrease in weight corresponds to the concentration of dry matter as water content diminishes [8]. Texturally, P32 was characterized by lower moisture content and a firm, dense structure, while Y25 and LK1 demonstrated higher moisture levels, resulting in a moist and tender texture. These findings offer valuable guidance for consumers in selecting appropriate sweet potato varieties for baking, depending on their texture preferences.

### 3.2. Electronic Nose and Electronic Tongue Analysis

**Figure 2:**
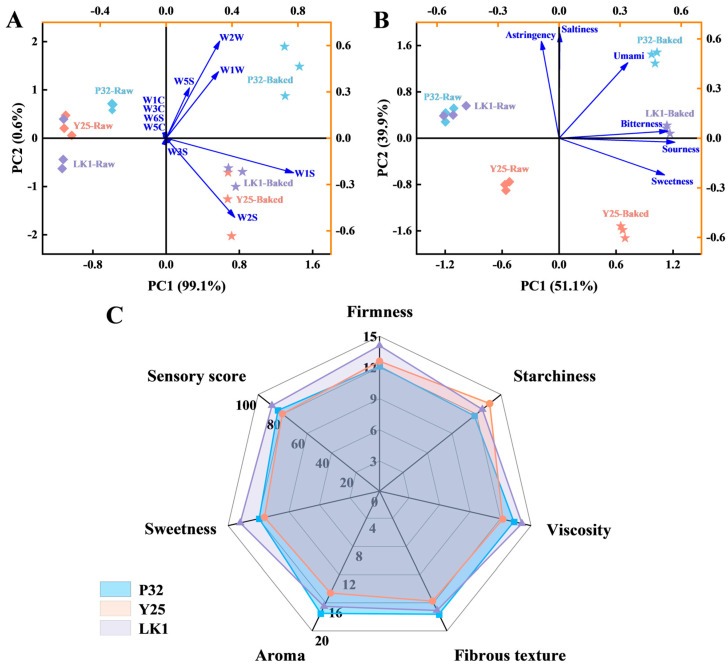
Flavour changes in different varieties of sweet potatoes before and after baking. (**A**) PCA of 10 volatile components by electronic nose method; (**B**) PCA analysis of 6 taste indicators by electronic tongue method; (**C**) Sensory scores of different varieties of baked sweet potatoes. P32: Pushu 32; Y25: Yanshu 25; LK1: Liankaoshu 1.

Principal component analysis (PCA) was used to evaluate the differences in aroma and taste of the three sweet potato varieties before and after baking. As shown in Figure 2A, the first principal component (PC1) explained 99.1% of the variance, while the second principal component (PC2) accounted for 0.6%, with a cumulative contribution of 99.7%, representing the majority of aroma information. Distinct differences in aroma characteristics were observed between raw and baked samples, as they were distributed on opposite sides of the PC1 axis. Compared to raw samples, baked sweet potatoes exhibited stronger responses on W1S, W2S, W2W, W1W, and W5S sensors, indicating that these volatile compounds significantly contributed to the baked aroma profile. Furthermore, signal intensities varied across varieties, with Y25 and LK1 samples displaying partial clustering. Notably, P32 samples consistently showed higher response values across the above sensors, both before and after baking, and were distinctly separated from the other two varieties, suggesting higher levels of alkanes, alcohols, aromatic compounds, sulfides, and nitrogen oxides, and thus more prominent aroma characteristics.

Figure 2B presents the PCA results of the electronic tongue analysis. PC1 and PC2 explained 51.1% and 39.9% of the variance, respectively, with a total contribution of 91.0%, reflecting the overall taste characteristics of the samples. All five taste attributes except astringency were positively correlated with PC1. Astringency, saltiness, umami, and bitterness were positively correlated with PC2, while sweetness and sourness were negatively correlated. Raw samples were located along the negative axis of PC1, farther from the taste sensor loadings, while baked samples were positioned on the positive axis, suggesting that PC1 is likely related to temperature and that baking significantly enhanced taste profiles. Notably, raw P32 and LK1 samples were closely clustered, but after baking, the taste profiles of the three varieties were clearly separated, highlighting their distinct taste characteristics. In addition, LK1 was closest to the sensor loadings, indicating the richest flavor characteristics, whereas Y25 showed weaker flavor signals, possibly due to its higher moisture content (Figure 1B).

### 3.3. Sensory Analysis

Sensory evaluation was conducted to assess the quality of baked sweet potatoes (Figure 2C). P32 exhibited a firm texture with high firmness and a noticeable starchy feel but low fibrousness, resulting in a smooth mouthfeel and pronounced aroma. Consequently, P32 scored lowest in firmness and starchy feel but highest in fibrous texture and aroma. In contrast, Y25, characterized by higher moisture content, had a soft and moist texture with slight fibrousness. However, its aroma and sweetness were relatively mild, leading to the highest score for starchy feel but the lowest for viscosity, fibrous texture, aroma, and sweetness. Although Y25 is generally considered a high-sweetness variety [12,14], its lower dry matter content in this study suggests insufficient starch levels, potentially reducing the production of reducing sugars during saccharification. Variations in agronomic practices and baking techniques, such as those optimized by Hou et al. [5] using response surface methodology, might also contribute to its suboptimal sensory performance.

LK1 exhibited a well-balanced sensory profile, characterized by fine texture, moderate aroma, optimal viscosity, and rich sweetness, leading to consistently high scores across multiple attributes. These results align with quality characteristics, electronic nose, and electronic tongue analyses, further underscoring LK1’s superior sensory properties. The overall sensory scores for P32, Y25, and LK1 were 83.7, 80.0, and 88.6, respectively, indicating that LK1 is the most appealing variety for consumers due to its harmonious combination of flavor, texture, and aroma.

### 3.4. VOCs Analysis

**Figure 3:**
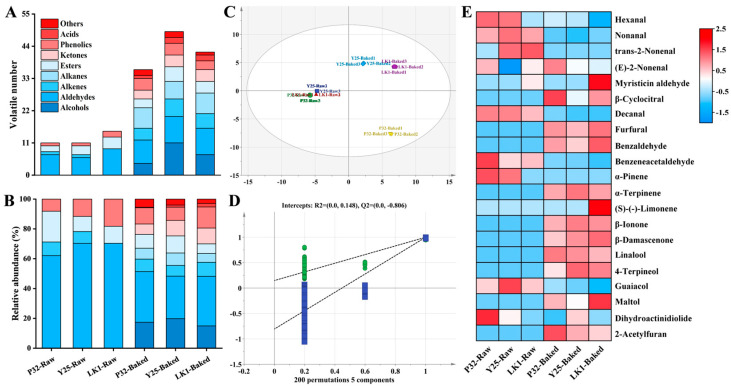
Changes in aroma composition of different varieties of sweet potatoes before and after baking. (**A**) Statistics of VOCs categories; (**B**) Trends in the relative content of VOCs; (**C**) OPLS-DA scoring plots; (**D**) Results of model cross-validation; and (**E**) Heatmap visualisation of key differential VOCs. Red colour indicates up-regulation of key differential VOCs and blue colour indicates down-regulation.

During the baking process, chemical reactions such as β-carotene degradation, caramelization, and Maillard browning generate key aromatic compounds, significantly influenced by varietal differences [35]. At high temperatures, organic compounds in sweet potatoes undergo pyrolysis or polymerization, resulting in more diverse VOCs [4]. As shown in Figure 3A, 11, 11, and 15 VOCs were identified in raw P32, Y25, and LK1 samples, respectively, while 36, 49, and 42 VOCs were identified in their baked counterparts. Across all samples, 70 VOCs were detected and classified into nine major chemical groups: alcohols (14), aldehydes (12), olefins (11), alkanes (10), esters (10), ketones (4), phenols (4), acids (3), and others (2) (Supplementary Table S3).

Alcohols, primarily derived from lipid oxidation and Strecker degradation [36], were detected exclusively in baked samples, with Y25 exhibiting the highest abundance (Figure 3B). Aldehydes are key aromatic compounds in sweet potatoes [37], typically formed via the auto-oxidation of unsaturated fatty acids and associated with fruity and green aromas [38]. They dominated the volatile profile of raw samples (62.09–70.31%) but significantly decreased after baking, likely due to accelerated lipid oxidation at high temperatures [39]. Notably, P32 retained a relatively high proportion of aldehydes (34.02%) post-baking. Alkenes, contributing fruity, spicy, and citrusy aromas [36], increased significantly post-baking, whereas alkanes were only detected in baked samples. Esters, formed by the interaction of alcohols and free fatty acids [40], generally decreased during baking, with P32 showing an 11.46% reduction. Ketones, generated from the oxidation of unsaturated fatty acids or the breakdown of alcohols and esters, contribute floral, fruity, and sweet aromas [41], and were only found in baked samples, with LK1 having the highest relative content (10.62%). Phenolics, associated with smoky aromas, increased in type and quantity post-baking but maintained stable relative abundances. Acids, contributing to cheese and citrus-like aromas, accumulated during baking but were often converted into alcohols or esters, limiting their proportion in the overall VOC profile. Acid content ranged from 0.33% to 2.36% among the three varieties.

To better distinguish the aroma characteristics of baked sweet potatoes, an orthogonal partial least squares discriminant analysis (OPLS-DA) model was constructed using VOCs as dependent variables and samples as independent variables (Figure 3C). The model’s reliability was evaluated using R^2^ and Q^2^ values, where values above 0.5 are considered acceptable. In this study, the R^2^x was 0.993, R^2^y was 0.999, and Q^2^ was 0.998. Additionally, the cross-validation results after 200 permutations (Figure 3D) showed a Q^2^ intercept below zero, indicating that the model was not overfitted. Notably, VOCs from raw and baked samples were distinctly separated along the PC1 axis, and baked Y25 and LK1 were distributed within the same quadrant, suggesting some similarity in their VOC profiles, consistent with the electronic nose results (Figure 2A).

Using *p* < 0.05 and variable importance projection (VIP) ≥ 1 as criteria, 21 key VOCs were identified, including 10 aldehydes, 3 olefins, 2 ketones, 2 alcohols, 2 phenolics, 1 ester, and 1 other compound. Hexanal, nonanal, and trans-2-nonenal, derived from fatty acid oxidation, contributed grassy, fatty, and green aromas and were enriched in raw samples, serving as key aroma contributors. Previous studies have identified benzaldehyde and benzeneacetaldehyde as important aroma compounds in sweet potatoes [21], noted for almond- and leafy-like freshness. Benzaldehyde is formed via the oxidation of benzyl alcohol [38] and showed greater accumulation in LK1, while P32 exhibited the highest benzeneacetaldehyde levels both before and after baking. During heating, β-carotene degradation produced β-cyclocitral and β-ionone, imparting woody, fruity, and fresh aromas to baked sweet potatoes [42]. Y25 exhibited higher levels of β-ionone, consistent with Shen et al. [13]. However, its overall abundance of key VOCs was lower, potentially explaining its lower aroma score. Tsai et al. [35] identified β-damascenone as a key aromatic compound in baked sweet potatoes, contributing floral and sweet notes due to its low threshold. Β-damascenone was detected in all baked samples, with LK1 showing the highest accumulation. Additionally, Maillard reaction products such as furfural, benzaldehyde, and maltol were significantly enriched in LK1, contributing caramel, bread, and almond aromas [7]. These high VOC levels enhanced LK1’s baked flavor. P32, enriched in benzeneacetaldehyde, β-cyclocitral, linalool, and 2-acetylfuran, presented floral, fruity, and caramel notes, making it the highest-rated variety for aroma.

### 3.5. Analysis of Soluble Sugar, Reducing Sugar, Sucrose, Maltose, Fructose, Glucose Contents, and Sweetness Value

**Figure 4:**
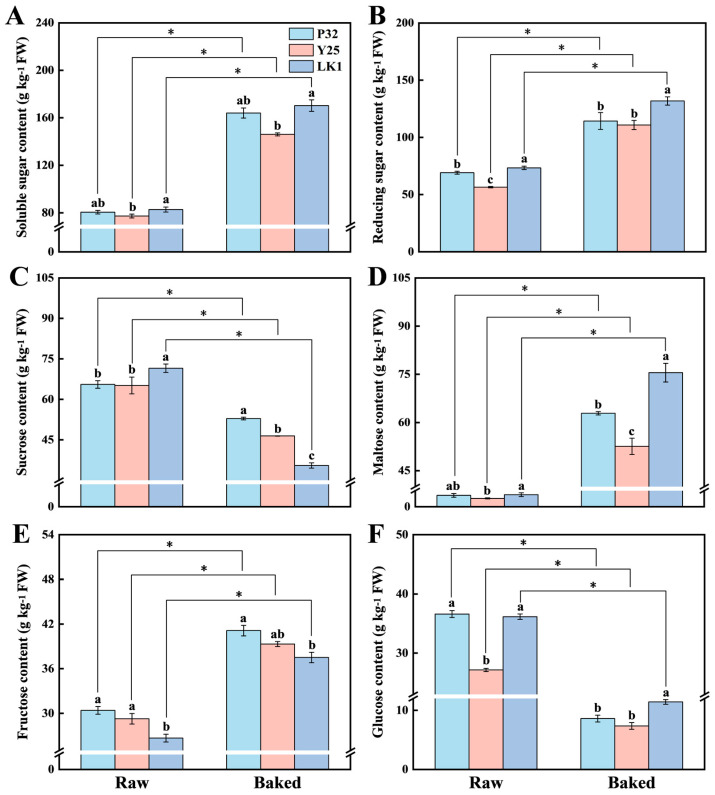
Soluble sugar (**A**), reducing sugar (**B**), sucrose (**C**), maltose (**D**), fructose (**E**), and glucose (**F**) contents of different varieties of sweet potatoes before and after baking. P32: Pushu 32; Y25: Yanshu 25; LK1: Liankaoshu 1. Data were presented as mean ± standard deviation, the varieties were analysed by ANOVA and different lower case letters indicate significant differences between varieties (*p* < 0.05); differences between raw and baked samples were assessed using an independent sample *t*-test, * indicates that there was a significant difference at *p* < 0.05.

The content and composition of soluble sugars are critical indicators of the sweetness characteristics of baked sweet potato varieties [43]. This study analyzed the changes in soluble sugar, reducing sugar, sucrose, maltose, fructose, and glucose contents before and after baking (Figure 4). As shown in Figure 4A,B, LK1 consistently exhibited the highest sugar content among the three varieties, while Y25 had the lowest levels (*p* < 0.05). Baking significantly increased the contents of soluble sugar and reducing sugar, consistent with the findings of Wei and Cao [43]. Specifically, after baking, the soluble sugar and reducing sugar contents in LK1 increased by 87.61 g kg^−1^ and 58.52 g kg^−1^, respectively.

According to Jiang et al. [7], sucrose is the dominant soluble sugar in raw sweet potatoes, but its content decreases significantly after baking (*p* < 0.05). In this study, sucrose levels in baked P32, Y25, and LK1 samples decreased by 12.64 g kg^−1^, 18.72 g kg^−1^, and 36.04 g kg^−1^, respectively, compared to their raw counterparts (Figure 4C, *p* < 0.05). Conversely, maltose content showed a marked increase after baking, reaching 62.87 g kg^−1^, 52.57 g kg^−1^, and 75.51 g kg^−1^ for P32, Y25, and LK1, respectively (Figure 4D, *p* < 0.05). Figure 4E,F illustrate that baking led to an increase in fructose content and a decrease in glucose content. This phenomenon can be attributed to the decomposition of sucrose into fructose and glucose during heating, with glucose subsequently participating in maltose synthesis [8]. Furthermore, during the early stages of heating, β-amylase activity cleaves starch molecules at non-reducing ends, producing substantial amounts of maltose [8,44].

It should be noted that the increase in soluble sugars and reducing sugars in baked sweet potatoes is primarily attributed to maltose accumulation [45,46]. Li et al. [12] emphasized that maltose formation during heating plays a decisive role in enhancing the sweetness of sweet potatoes. In this study, the sweetness values of P32 and LK1 were significantly higher than that of Y25, exceeding it by 22.40 and 20.59, respectively (Supplementary Figure S1, *p* < 0.05), which may be partially due to the relatively lower maltose content in Y25. Combined with the results from the electronic tongue and sensory evaluation (Figure 2B,C), LK1 demonstrated excellent performance in both sweetness and saccharification efficiency, indicating that it is a high-quality sweet potato variety well-suited for baking.

### 3.6. Analysis of Starch and Amylose Content

**Figure 5:**
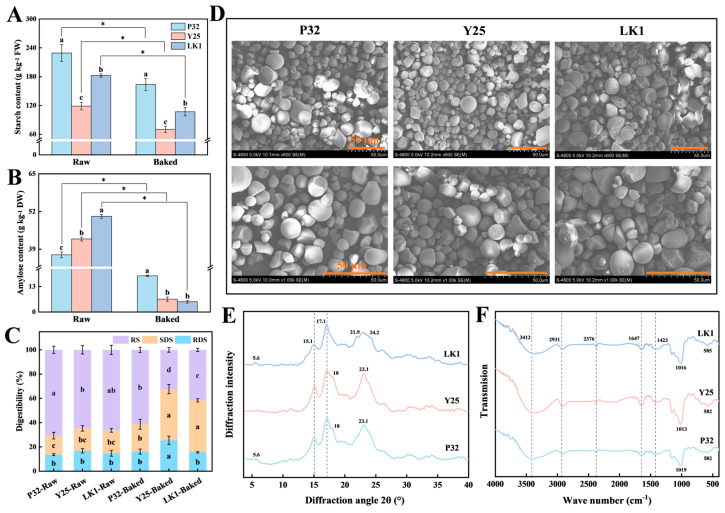
Starch (**A**) and amylose (**B**) contents of different varieties of sweet potatoes before and after baking. Data were presented as mean ± standard deviation, the varieties were analysed by ANOVA and different lower case letters indicate significant differences between varieties (*p* < 0.05); differences between raw and baked samples were assessed using an independent sample *t*-test, * indicates that there was a significant difference at *p* < 0.05. In vitro digestive characteristics of starch from different varieties of sweet potatoes before and after baking (**C**). Data were presented as mean ± standard deviation, the samples were analysed by ANOVA and different lower case letters indicate significant differences between samples (*p* < 0.05). Observation of morphological properties of starch granules (**D**) (scale bar: 50 μm), XRD diffractogram (**E**), and FT-IR spectra (**F**). P32: Pushu 32; Y25: Yanshu 25; LK1: Liankaoshu 1.

The composition and content of starch are pivotal factors affecting the texture and taste of baked sweet potatoes [8,22]. As depicted in Figure 5A, P32 consistently exhibited the highest starch content both before and after baking, followed by LK1, while Y25 displayed the lowest levels. Under the combined influence of high temperatures and amylase activity, starch underwent hydrolysis and gelatinization, converting into soluble sugars [15,47]. Baking significantly reduced starch content (*p* < 0.05), with decreases of 65.40 g kg^−1^, 48.45 g kg^−1^, and 75.30 g kg^−1^ for P32, Y25, and LK1, respectively. These reductions corresponded to percentage decreases of 28.52%, 40.79%, and 41.29%.

Distinct changes in amylose content were also observed before and after baking (Figure 5B). In raw samples, P32 exhibited the lowest amylose content, while LK1 had the highest. However, after baking, this trend reversed, with Y25 and LK1 showing substantial decreases of 84.62% and 89.61%, respectively, while P32 displayed a relatively smaller reduction of only 50.00%. Devi et al. [48] reported that rice varieties with higher amylose content often exhibit greater firmness. In this study, the relatively higher levels of starch and amylose in baked P32 may explain its firmer texture, higher viscosity, and more pronounced starchy mouthfeel.

### 3.7. Analysis of Starch In Vitro Digestibility

Starch, a major nutritional component in sweet potatoes, is digested in the human gastrointestinal system to release glucose for absorption. Depending on its digestion rate, starch is classified into RDS, SDS, and RS [49]. As illustrated in Figure 5C, raw samples from all three sweet potato varieties exhibited comparable proportions of RDS, SDS, and RS. However, baking significantly increased the SDS proportion while reducing the RS proportion (*p* < 0.05). Among the varieties, Y25 showed the most substantial changes, with RDS and SDS proportions increasing by 8.59% and 23.75%, respectively, and RS decreasing by 32.34%.

Significant differences in starch digestibility were observed among the three varieties. After baking, Y25 exhibited higher levels of RDS and SDS, which are beneficial for providing immediate energy. However, due to their potential impact on blood glucose regulation and weight management, Y25 may have limited suitability for individuals with diabetes or those aiming for weight control [50]. In contrast, P32 retained a higher level of RS, indicating greater resistance to digestion and suggesting potential health benefits for consumers with hyperglycemia or insulin resistance [51]. These findings highlight the nutritional diversity of baked sweet potatoes and offer valuable insights for dietary planning and consumer health.

### 3.8. Observation of Starch Morphology and Particle Size Distribution

Starch is a semi-crystalline granular composed of alternating amorphous and semi-crystalline growth rings, with significant differences in shape, size, and crystal type among sweet potato varieties [52]. According to Allan, Read, and Johanningsmeier [15], starch content and crystal proportion are critical factors in predicting the sensory attributes of baked sweet potatoes. As illustrated in Figure 5D, starch granules from all three varieties exhibited similar morphologies, being predominantly round or oval, with some granules appearing polygonal or hemispherical. The granule surfaces were smooth, with no visible cracks or pores, consistent with previous findings by Chen et al. [53] and Gou et al. [54]. However, the particle size of starch differed significantly between varieties.

Further analysis of starch particle size and distribution characteristics (Table 1) revealed that LK1 had the largest values for *D*_10_, *D*_50_, *D*_90_, *D*_(4,3)_, and *D*_(3,2)_, followed by P32, with Y25 having the smallest values. In contrast, S.S.A. exhibited the opposite trend. Additionally, LK1 had a significantly lower dispersion value compared to P32 and Y25, indicating a more uniform particle size distribution. While Y25 and LK1 exhibited considerable differences in starch particle size, both varieties displayed similarly soft and fine textures after baking. This suggests that particle size alone does not determine texture. Similarly, Allan et al. [15] concluded that starch particle size is not a strong predictor of baked sweet potato texture.

### 3.9. Crystalline Structure Analysis of Starch

The crystalline structure of starch is a key determinant of its physicochemical properties and is generally classified into A-, B-, and C-types, each exhibiting distinct diffraction peak intensities and positions [55]. The XRD pattern reflects the crystal structure of the starch [56]. As depicted in Figure 5E, the XRD patterns of the three sweet potato varieties revealed significant differences. Y25 starch exhibited prominent single peaks at 15.1° and 23.1° and a double peak between 17.1° and 18°, indicating a typical A-type crystalline structure [54]. LK1 starch showed distinct single peaks at 5.6°, 15.1°, and 17.1°, along with double peaks at 21.9° and 24.2°, characteristic of a B-type crystalline structure [49]. The diffraction pattern of P32 starch, featuring peaks intermediate between those of LK1 and Y25, demonstrated characteristics of both A- and B-type crystals, consistent with C-type starch [57]. Further analysis indicated that C-type starch can be subclassified into CA-, CB-, and CC-types based on the ratio of A- to B-type crystals. The shoulder peak observed at 18° in the XRD pattern of P32 identified its crystalline type as CA-type [58].

According to Allan et al. [22], increasing the proportion of A-type crystalline starch reduces enzymatic hydrolysis sensitivity during heating, preserving the structural integrity of starch molecules and limiting maltose production. Conversely, B-type crystalline starch, which has a lower gelatinization temperature and requires a longer gelatinization time, contributes to a more pronounced sweet flavor during baking. This may explain why Y25 exhibits a lower maltose content while LK1 displays higher sweetness levels. Furthermore, the crystalline structure of sweet potato starch is not solely determined by genotype but is also influenced by external factors and environmental conditions [16,59].

### 3.10. Molecular Structure Analysis of Starch

FT-IR spectroscopy provides functional group information at specific wavelengths [60]. As shown in Figure 5F, all samples exhibited characteristic absorption peaks at 3412, 2931, 1647, and 1423 cm^−1^, corresponding to O-H stretching, C-H stretching, H-O-H bending, and C-H deformation or symmetric stretching vibrations, respectively [34,61]. These findings indicate a high degree of similarity in the chemical structure of starch across the three varieties. However, the Y25 sample showed a stronger absorption peak within the 3000–3600 cm^−1^ range, suggesting a higher content of hydroxyl (-OH) groups. Additionally, the C-O stretching vibration observed at 1013–1019 cm^−1^ reflects the presence of ether bonds, while the absorption peaks at 582–585 cm^−1^ (C-C and C-O-C bending vibrations) confirm the molecular backbone of starch. Overall, the positions and shapes of the characteristic absorption peaks were similar among the three varieties, with slight differences likely caused by genetic variations, leading to subtle modifications in the starch molecular structure without the formation of new functional groups or chemical bonds.

The absorption peaks at 1047 cm^−1^ and 1022 cm^−1^ represent the crystalline and amorphous regions of starch, respectively [62]. Additionally, the absorption peak at 995 cm^−1^ is highly sensitive to moisture content and reflects the single-helix crystalline structure [34,63]. R_1047/1022_ is commonly used to evaluate the short-range ordering of starch, while R_995/1022_ quantifies the spatial arrangement of double-helix structures within the crystalline region [64]. In this study, R_1047/1022_ for P32, Y25, and LK1 ranged from 1.094 to 1.239 (Table 1), and R_995/1022_ values were 0.980, 1.067, and 1.023, respectively. These results suggest that Y25 has a slightly higher proportion of ordered structures, whereas P32 exhibits a more stable double-helix crystalline arrangement.

### 3.11. Spearman Correlation Analysis

**Figure 6:**
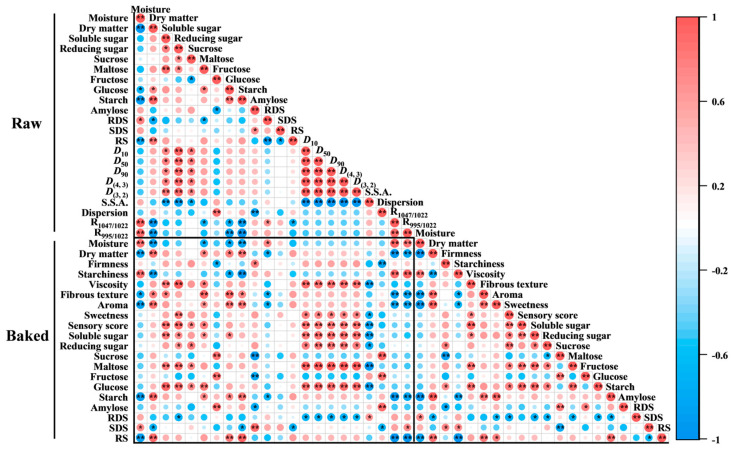
Spearman’s correlation heatmap for raw and baked samples. * indicates significant correlation at *p* < 0.05, and ** indicates highly significant correlation at *p* < 0.01. Red represents different degrees of positive correlation and blue represents different degrees of negative correlation, as shown in the scale bar on the right side of the heat map.

To elucidate the mechanisms by which the physicochemical properties of sweet potatoes, starch characteristics, and changes in multiscale structures affect the sensory quality of baked sweet potatoes, Spearman correlation heatmaps were constructed (Figure 6). Notably, distinct differences in the overall correlation patterns across various regions were observed, indicating that baking significantly altered the relationships among these indicators. Specifically, the starch content in raw sweet potatoes exhibited a very significant negative correlation (*p* < 0.01) with the moisture content and starchiness of the baked samples, while showing significant (*p* < 0.05) or very significant (*p* < 0.01) positive correlations with fibrous texture and aroma. These findings suggest that the interaction between water and starch intensifies during gelatinization, and starch degradation is intricately linked not only to saccharification reactions but also to changes in texture and aroma, thereby enhancing the flavor and texture of baked sweet potatoes.

Moreover, strong positive correlations were identified between starch particle size (*D*_10_, *D*_50_, *D*_90_, *D*_(4,3)_, and *D*_(3,2)_) and the viscosity, sweetness, sensory scores, soluble sugar, reducing sugar, and maltose content of baked sweet potatoes. These results indicate that starch particle size distribution is a critical factor controlling the saccharification process at high temperatures, playing an essential role in flavor development during baking. Additionally, sweetness demonstrated significant (*p* < 0.05) or very significant (*p* < 0.01) positive correlations with sensory scores, soluble sugar, reducing sugar, maltose, and glucose contents, underscoring that sweetness is a key factor influencing consumer preference for baked sweet potatoes. Higher sweetness levels correspond to greater consumer appeal. In summary, the sensory quality of baked sweet potatoes arises from a complex interplay of starch physicochemical properties, moisture loss, starch degradation, and saccharification processes during baking.

## 4. Conclusions

This study provides a comprehensive analysis of the flavor and starch physicochemical properties of three baked sweet potato varieties (P32, Y25, and LK1) cultivated in northeastern China, revealing significant differences in quality attributes, flavor profiles, and sensory scores. LK1 exhibited the highest sweetness and overall sensory score, attributed to its superior saccharification efficiency, accumulation of key volatile compounds such as β-damascenone and maltol, and a fine, balanced texture. P32, with its firm texture and distinctive aroma, was characterized by its higher starch and amylose content and unique volatile profile, including linalool and 2-acetylfuran. In contrast, Y25 displayed a moist and tender texture, but its lower sugar content and sensory scores highlight its appeal to specific consumer preferences. Baking significantly induced physicochemical changes in starch, including reductions in starch and amylose content and an increase in saccharification levels, which collectively shaped the sensory quality and flavor of baked sweet potatoes. Furthermore, Spearman correlation analysis revealed that starch particle size and saccharification efficiency were critical factors influencing sweetness and sensory attributes. Overall, this study underscores the pivotal role of starch properties and saccharification in determining the flavor and texture of baked sweet potatoes, offering valuable insights into variety selection, breeding, and process optimization, while providing practical guidance for food product development and meeting diverse consumer demands.

## Figures and Tables

**Table 1:** Particle size distribution and FT-IR ratios of sweet potato starch from different varieties.

	*D*_10_ (μm)	*D*_50_ (μm)	*D*_90_ (μm)	*D*_(4,3)_ (μm)	*D*_(3,2)_ (μm)	S.S.A. (m^2^ kg^−1^)	Dispersion	R_1047/1022_	R_995/1022_
P32	7.60 ± 0.00 ^B^	18.37 ± 0.01 ^B^	36.86 ± 0.03 ^B^	20.49 ± 0.02 ^B^	9.91 ± 0.00 ^B^	224.13 ± 0.21 ^B^	1.593 ± 0.003 ^A^	1.094 ± 0.001 ^C^	0.980 ± 0.001 ^B^
Y25	5.25 ± 0.00 ^C^	14.05 ± 0.01 ^C^	27.59 ± 0.06 ^C^	15.86 ± 0.03 ^C^	7.79 ± 0.00 ^C^	285.03 ± 0.15 ^A^	1.590 ± 0.002 ^A^	1.239 ± 0.004 ^A^	1.067 ± 0.021 ^A^
LK1	10.47 ± 0.01 ^A^	22.08 ± 0.02 ^A^	39.99 ± 0.04 ^A^	23.82 ± 0.02 ^A^	12.72 ± 0.01 ^A^	174.63 ± 0.12 ^C^	1.337 ± 0.005 ^B^	1.165 ± 0.002 ^B^	1.023 ± 0.023 ^A^

Note: *D*_(4,3)_, particle diameter of volume; *D*_(3,2)_, particle diameter of surface; S.S.A., specific surface of unit volume; the value of dispersion calculated as (*D*_90_ − *D*_10_)/*D*_50_; *D*_10_, *D*_50_, and *D*_90_ represent the corresponding particle size which is smaller than 10%, 50%, and 90% of the sample particles, respectively. R_1047/1022_ and R_995/1022_, ratios of infrared absorbance at 1047/1022 cm^−1^ and 995/1022 cm^−1^; P32: Pushu 32; Y25: Yanshu 25; LK1: Liankaoshu 1. Data were analysed by ANOVA, and different capital letters indicate significant differences between samples (*p* < 0.05).

## Data Availability

The original contributions presented in this study are included in the article and Supplementary Material. Further inquiries can be directed to the corresponding author.

## Supplementary Materials

The following supporting information can be downloaded at: https://www.mdpi.com/article/10.3390/foods15050802/s1, Figure S1: Sweetness values of different varieties of sweet potato before and after baking. Data were presented as mean ± standard deviation, the varieties were analysed by ANOVA and different lower case letters indicate significant differences between varieties (*p* < 0.05); differences between raw and baked samples were assessed using an independent sample *t*-test, * indicates that there was a significant difference at *p* < 0.05, while ns indicates that there was no significant difference; Table S1: Standard sensor arrays and performance specification in electronic nose PEN 3; Table S2: Sensory evaluation standards; Table S3: Volatile compounds identified in different samples by GC-MS.
