# Supplementary material for: Comparative Analysis of Flavor and Starch Physicochemical Properties in Different Varieties of Baked Sweet Potatoes
Source: Foods. 2026 Feb 24;15(5):802. doi: 10.3390/foods15050802 (PMC12984904; doi:10.3390/foods15050802)
Supplement: Supplementary file 1 [file foods-15-00802-s001.zip › Table S2.docx]

**Table S2.** Sensory evaluation standards

| Sensory properties | Total score | Standards | Score |
| --- | --- | --- | --- |
| Firmness | 15 | The flesh of sweet potato has the right amount of softness and is easy to chew | 11–15 |
|  |  | Sweet potato flesh is soft or hard, average chewiness | 6–10 |
|  |  | Sweet potato flesh too soft or too hard, poor chewing type | 1–5 |
| Starchiness | 15 | Sweet potato flesh is palatable, with no obvious starchy texture | 11–15 |
|  |  | Sweet potato flesh is moderately palatable and has a somewhat starchy texture | 6–10 |
|  |  | Poor palatability of sweet potato flesh, with obvious starchy texture | 1–5 |
| Viscosity | 15 | Sweet potato flesh with appropriate viscosity and high acceptability | 11–15 |
|  |  | Sweet potato flesh viscosity is high or low, acceptance is average | 6–10 |
|  |  | Sweet potato flesh viscosity is too high or too low for poor acceptance | 1–5 |
| Fibrous texture | 15 | The flesh of the sweet potato is not obviously fibrous and has a smooth texture | 11–15 |
|  |  | Sweet potato flesh has less fibre and moderate texture | 6–10 |
|  |  | Sweet potato flesh with more crude fibre and poor taste | 1–5 |
| Aroma | 20 | Strong aroma of baked sweet potato | 16–20 |
|  |  | Baked sweet potato is moderately aromatic | 11–15 |
|  |  | Baked sweet potato has a mild aroma or a slightly mushy flavour | 6–10 |
|  |  | Basically no baked sweet potato aroma or heavy paste flavour | 1–5 |
| Sweetness | 20 | Has a rich sweet baked sweet potato flavour | 16–20 |
|  |  | Baked sweet potato is moderately sweet | 11–15 |
|  |  | Baked sweet potato is less sweet or slightly tart | 6–10 |
|  |  | Baked sweet potato with very little sweetness or a strong acidic flavour | 1–5 |
