# Supplementary material for: Comparative Analysis of Flavor and Starch Physicochemical Properties in Different Varieties of Baked Sweet Potatoes
Source: Foods. 2026 Feb 24;15(5):802. doi: 10.3390/foods15050802 (PMC12984904; doi:10.3390/foods15050802)
Supplement: Supplementary file 1 [file foods-15-00802-s001.zip › Table S3.docx]

Table S3 Volatile compounds identified in different samples by GC-MS

| No. | Compound (quantity) | CAS Number | Area Percentage % | | | | | |
| --- | --- | --- | --- | --- | --- | --- | --- | --- |
|  |  |  | P32-Raw | Y25-Raw | LK1-Raw | P32-Baked | Y25-Baked | LK1-Baked |
|  | **Alcohols (14)** |  |  |  |  |  |  |  |
| 1 | Linalool | 78-70-6 | - | - | - | 6.32 | 5.85 | 4.87 |
| 2 | 3,4-DiMethylcyclohexanol | 5715-23-1 | - | - | - | 2.59 | 0.85 | 0.79 |
| 3 | 2,2-dimethylhexan-1-ol | 2370-13-0 | - | - | - | 4.55 | - | - |
| 4 | 4-Ethyl-1-octyn-3-ol | 5877-42-9 | - | - | - | - | - | 1.17 |
| 5 | 2,6-DiMethylcyclohexanol | 5337-72-4 | - | - | - | - | 0.79 | 1.08 |
| 6 | 4-Terpineol | 562-74-3 | - | - | - | 3.94 | 7.13 | 6.39 |
| 7 | 2-(4-Methylphenyl)propan-2-ol | 1197-01-9 | - | - | - | - | 0.87 | - |
| 8 | 2,2-Dimethyl octanol | 2370-14-1 | - | - | - | - | 0.70 | - |
| 9 | Trans-2-Tridecen-1-ol | 74962-98-4 | - | - | - | - | 1.23 | - |
| 10 | 2-Octanol | 123-96-6 | - | - | - | - | 0.35 | - |
| 11 | 2,2,4-trimethylpentan-1-ol | 123-44-4 | - | - | - | - | - | 0.28 |
| 12 | 4,8-Dimethyl-1,7-nonadien-4-ol | 17920-92-2 | - | - | - | - | 0.69 | - |
| 13 | (1R,2R,3S,5R)-(-)-2,3-Pinanediol | 22422-34-0 | - | - | - | - | 0.35 | - |
| 14 | 2-ethyl-4-methylpentan-1-ol | 106-67-2 | - | - | - | - | 0.97 | 0.40 |
|  | **Aldehydes (12)** |  |  |  |  |  |  |  |
| 15 | Nonanal | 124-19-6 | 17.61 | 21.46 | 18.24 | 3.93 | 2.39 | 4.95 |
| 16 | Decanal | 112-31-2 | 16.93 | 16.87 | 14.59 | 7.63 | 6.45 | 6.37 |
| 17 | (E)-2-Nonenal | 18829-56-6 | 4.12 | - | 3.44 | 4.78 | 3.14 | 2.85 |
| 18 | trans-2-Nonenal | 18829-56-6 | 2.13 | 16.98 | 18.36 | - | - | - |
| 19 | Furfural | 98-01-1 | - | - | - | 4.22 | 3.52 | 4.78 |
| 20 | Hexanal | 66-25-1 | 7.43 | 7.16 | 2.63 | 3.17 | 2.81 | - |
| 21 | (E)-2-Octenal | 2548-87-0 | - | - | 1.82 | - | 3.77 | - |
| 22 | β-Cyelocitral | 432-25-7 | - | - | - | 4.25 | 1.36 | 3.13 |
| 23 | Tetradecanal | 124-25-4 | 1.25 | - | 1.11 | - | - | 0.43 |
| 24 | Myristicin aldehyde | 5780-07-4 | - | - | 1.65 | - | - | 5.08 |
| 25 | Benzaldehyde | 100-52-7 | - | - | - | 2.86 | 2.23 | 3.69 |
| 26 | Benzeneacetaldehyde | 122-78-1 | 12.62 | 7.84 | 8.56 | 3.18 | 2.91 | 1.94 |
|  | **Terpenes (11)** |  |  |  |  |  |  |  |
| 27 | α-Pinene | 80-56-8 | 9.17 | 7.92 | - | - | - | - |
| 28 | d-Longifolene | 475-20-7 | - | - | - | 1.65 | - | - |
| 29 | α-Humulene | 6753-98-6 | - | - | - | 2.37 | - | - |
| 30 | α-longipinene | 5989-08-2 | - | - | - | 1.24 | - | 0.51 |
| 31 | (S)-(-)-Limonene | 5989-54-8 | - | - | - | - | - | 4.94 |
| 32 | (+)-Aromadendrene | 489-39-4 | - | - | - | - | 0.77 | 0.37 |
| 33 | α-Terpinene | 99-86-5 | - | - | - | 3.09 | 3.80 | 3.19 |
| 34 | Terpinolene | 586-62-9 | - | - | - | - | 0.57 | - |
| 35 | Valencene | 4630-07-3 | - | - | - | - | 1.05 | - |
| 36 | (+)-Limonene | 5989-27-5 | - | - | - | - | 0.65 | - |
| 37 | Elemene | 33880-83-0 | - | - | - | - | 0.35 | 0.42 |
|  | Alkanes (10) |  |  |  |  |  |  |  |
| 38 | Hexadecane | 544-76-3 | - | - | - | 1.38 | 0.93 | 1.10 |
| 39 | Tridecane | 629-50-5 | - | - | - | 0.65 | - | 1.18 |
| 40 | Heptadecane | 629-78-7 | - | - | - | 0.45 | 1.44 | 0.50 |
| 41 | Tetracosane | 646-31-1 | - | - | - | 0.63 | 1.59 | - |
| 42 | 3,6-Dimethyloctane | 15869-94-0 | - | - | - | 1.79 | 1.95 | 0.26 |
| 43 | Eicosane | 112-95-8 | - | - | - | 0.93 | - | - |
| 44 | Pentadecane | 629-62-9 | - | - | - | 1.45 | 1.35 | 0.80 |
| 45 | Tetradecane | 629-59-4 | - | - | - | - | 1.03 | - |
| 46 | Nonadecane | 629-92-5 | - | - | - | - | - | 0.96 |
| 47 | 5-(2-Methylpropyl)nonane | 62185-53-9 | - | - | - | - | - | 1.16 |
|  | **Esters (10)** |  |  |  |  |  |  |  |
| 48 | Octadecanoic acid | 112-61-8 | - | - | - | 1.60 | - | - |
| 49 | Ethyl palmitate | 628-97-7 | - | - | - | 1.52 | - | - |
| 50 | Ethyl Stearate | 111-61-5 | - | - | - | 3.89 | - | - |
| 51 | Dibutyl phthalate | 84-74-2 | - | - | - | - | 0.96 | 0.52 |
| 52 | Diisobutyl phthalate | 84-69-5 | - | - | - | - | 0.86 | 0.97 |
| 53 | Bis(2-ethylhexyl) adipate | 103-23-1 | - | 2.09 | 0.54 | - | 0.82 | - |
| 54 | Methyl 2-hydroxyisobutyrate | 2110-78-3 | - | - | 0.41 | - | - | - |
| 55 | Dihydroactinidiolide | 15356-74-8 | 13.05 | 7.00 | 3.55 | 2.34 | 8.08 | 3.83 |
| 56 | 1,2-Benzenedicarboxylicacid, b | 85-69-8 | - | 1.12 | - | - | 0.74 | 1.05 |
| 57 | 2,2,4-trimethyl-1,3-pentanedio | 6846-50-0 | 7.55 | - | 6.96 | - | - | - |
|  | **Ketones (4)** |  |  |  |  |  |  |  |
| 58 | 7H-2,4a-Methanonaphthalen-7-on | 26839-52-1 | - | - | - | 0.27 | 0.63 | 0.64 |
| 59 | β-Ionone | 79-77-6 | - | - | - | 3.24 | 4.03 | 3.74 |
| 60 | 3-Eicosanone | 2955-56-8 | - | - | - | - | 1.34 | 1.11 |
| 61 | β-Damascenone | 23726-93-4 | - | - | - | 3.38 | 4.44 | 5.13 |
|  | **Phenols (4)** |  |  |  |  |  |  |  |
| 62 | 3,5-Di-tert-butylphenol | 1138-52-9 | - | - | 9.85 | 0.41 | 1.08 | 2.69 |
| 63 | 2-Methoxy-4-vinylphenol | 7786-61-0 | - | - | - | 0.31 | 0.40 | - |
| 64 | Maltol | 118-71-8 | - | - | - | 5.14 | 3.22 | 8.50 |
| 65 | Guaiacol | 90-05-1 | 8.14 | 11.57 | 8.34 | 5.05 | 4.17 | 2.96 |
|  | **Acids (3)** |  |  |  |  |  |  |  |
| 66 | Nonanoic acid | 112-05-0 | - | - | - | 0.33 | 0.90 | 0.99 |
| 67 | Decanoic acid | 334-48-5 | - | - | - | - | 0.49 | - |
| 68 | 2-ethylexanoic acid | 149-57-5 | - | - | - | - | - | 1.37 |
|  | Others (2) |  |  |  |  |  |  |  |
| 69 | 2-Acetylfuran | 1192-62-7 | - | - | - | 5.15 | 3.56 | 2.92 |
| 70 | Dibutylformamide | 761-65-9 | - | - | - | 0.32 | 0.44 | - |

Note: Compounds marked in red font are the selected major compounds; those marked in black font are minor compounds.
