# Supplementary material for: Comparative Analysis of Flavor and Starch Physicochemical Properties in Different Varieties of Baked Sweet Potatoes
Source: Foods. 2026 Feb 24;15(5):802. doi: 10.3390/foods15050802 (PMC12984904; doi:10.3390/foods15050802)
Supplement: Supplementary file 1 [file foods-15-00802-s001.zip › Figure S1.docx]

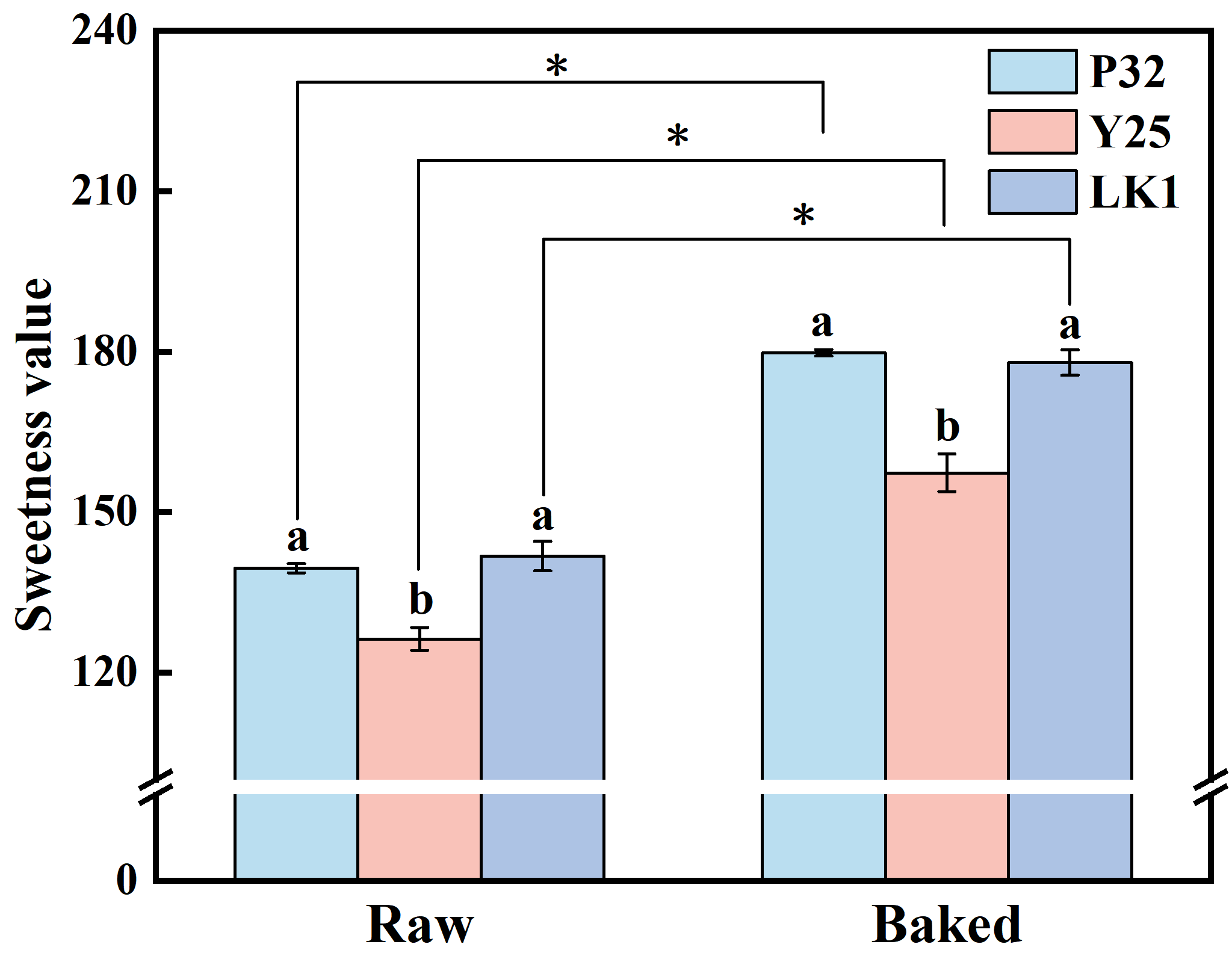


**Fig. S1** Sweetness values of different varieties of sweet potato before and after baking. Data were presented as mean ± standard deviation, the varieties were analysed by ANOVA and different lower case letters indicate significant differences between varieties (*P* < 0.05); differences between raw and baked samples were assessed using an independent sample T-test, * indicates that there was a significant difference at *P* < 0.05, while ns indicates that there was no significant difference.
