# Supplementary material for: Comparative Analysis of Flavor and Starch Physicochemical Properties in Different Varieties of Baked Sweet Potatoes
Source: Foods. 2026 Feb 24;15(5):802. doi: 10.3390/foods15050802 (PMC12984904; doi:10.3390/foods15050802)
Supplement: Supplementary file 1 [file foods-15-00802-s001.zip › Table S1.docx]

**Table S1.** Standard sensor arrays and performance specification in electronic nose PEN 3

| Sensor number | Sensor name | Performance specification |
| --- | --- | --- |
| S1 | W1C | Aromatic components |
| S2 | W5S | Nitrogen oxides |
| S3 | W3C | Ammonia water and aromatic components |
| S4 | W6S | Hydrogen |
| S5 | W5C | Aromatic aliphatics |
| S6 | W1S | Broad methane |
| S7 | W1W | Sulfur components |
| S8 | W2S | Alcohols |
| S9 | W2W | Aromatic components and organic sulfides |
